# Understanding the prolonged impact of online sexual abuse occurring in childhood

**DOI:** 10.3389/fpsyg.2023.1281996

**Published:** 2023-10-24

**Authors:** Felipa Schmidt, Filippo Varese, Sandra Bucci

**Affiliations:** ^1^Division of Psychology and Mental Health, The University of Manchester, Manchester, United Kingdom; ^2^Complex Trauma and Resilience Unit, Greater Manchester Mental Health Foundation Trust, Manchester, United Kingdom

**Keywords:** sexual abuse, technology, mental health, adverse childhood experiences, trauma, online harms

## Abstract

**Introduction:**

There has been a rapid increase in prevalence rates of online sexual abuse (OSA). Existing research has highlighted the negative impact OSA can have on victims. However, there is a gap in understanding the long-term impact of OSA when it occurred in childhood.

**Methods:**

This qualitative study comprised interviews with eight female participants aged 18–28  years recruited in UK NHS Trusts, and via mental health charities, University bulletins and social media. Each participant self-reported having experienced abuse through either the production or dissemination of sexual material online.

**Results:**

Results showed that the longer-term impact of OSA was multi-fold, including negative impact on sense of self and broader interpersonal relationships, and significant impact on the participants’ mental health, including experiences of self-harm, anxiety, and low mood. Likewise, participants discussed long-term apprehension to taking images and the added fear and worry that their sexual images were distributed online. Seven participants had received mental health support but only two recounted a positive experience when receiving support for OSA.

**Discussion:**

Future research using a quantitative longitudinal design is needed to further explore the prolonged impact of OSA. Clinical implications of the research highlight the need for support services to assess the impact of OSA and interventions that target OSA experiences.

## 1. Introduction

Increased internet usage has also brought an increased risk in online harms. These harms include online sexual harms and online sexual abuse (OSA). The latter can be understood as any type of sexual abuse with an online element, such as online grooming or abuse through the production or dissemination of sexual images or videos. Data by UK charity the National Society for the Prevention of Cruelty to Children (NSPCC) found that online grooming has increased by 80% from 2017/18 to 2021/22 (NSPCC, 2022). Between 2021/2022, the Internet Watch Foundation (2023), a UK organization assessing online child sexual abuse material (CSAM), noted a 4% increase CSAM reports, totaling 375,230 reports. This included a 9% increase of self-generated sexual abuse material, which includes material that individuals are coerced into taking by an offender (Internet Watch Foundation, 2023).

The impact of offline child sexual abuse (CSA) has been extensively studied. Existing research has shown that victims experience long-term mental and physical health challenges, as well as difficulties in adult interpersonal relationships (Davis and Petretic-Jackson, 2000; Fisher et al., 2017; Downing et al., 2021; Assini-Meytin et al., 2022). Compared with offline CSA, the distinguishable characteristics of OSA are its global reach, accessibility, and the permanence associated with the internet (Beech et al., 2008; Whittle et al., 2013a; Martin, 2015; Hanson, 2017; Forni et al., 2020), which can often perpetuate a sense fear, anxiety, and embarrassment for the victim (Martin, 2015; Hanson, 2017). Victims who become aware that their images and videos have circulated online report experiencing pervasive feelings of threat (Hanson, 2017). Additionally, permanence that the internet brings can mean that there is often no definable end to the abuse, resulting in re-traumatization of the abusive event. Victims of online grooming describe the manipulation and coercion that they experienced can often lead to feelings of shame and self-blame (Hanson, 2017). This can be exacerbated when a victim is coerced into taking and sending sexual material to an offender and when they discover that images are non-consensually distributed online (Martin, 2015).

Much like offline CSA, victims of OSA often do not disclose the abuse until years later. In fact, in many cases, OSA is discovered through law enforcement investigations (Svedin and Back, 2011; Whittle et al., 2013a, 2015; Martin, 2015), rather than victim disclosure. In cases of online grooming, victims often do not disclosure out of fear of the offender finding them, or they have been shamed into silence (Martin and Slane, 2015). Further, victims of OSA may not believe that the abuse is severe enough or that their experience can even be categorized as abuse if there is no physical or offline element (Quayle et al., 2012).

Existing research exploring how OSA affects an individual during adulthood is nascent. There is a specific lack of research that explores the journey of impact; from the immediate impact following the abusive event to the long-term impacts in adulthood. Likewise, the delayed impact of, and support offered for, these specific experiences from the victim’s perspective is not well understood. Qualitative interviews are an established methodological approach when exploring novel research areas (Newman and Kaloupek, 2004; Rossetto, 2014) and provide an important foundational understanding of an area to inform future research. Therefore, the aims of this study were to use qualitative methods to explore: (a) short and long-term psychological impact of online grooming and abuse through the taking and sharing of sexual images and videos occurring in childhood; and (b) victim perceptions of barriers.

## 2. Materials and methods

### 2.1. Participants

The study recruited participants (*N* = 8) through convenience sampling. Participants were recruited through either Child and Adolescent Mental Health Services (CAMHS) or a Sexual Assault Referral Centre from three National Health Service (NHS) Trusts in the Northwest of England. Recruitment also took place through nation-wide charity organizations, focused on providing mental health support or supporting victims of online/offline abuse, social media and advertisements posted on university bulletins. Participants were eligible if they: (a) self-reported experiencing online grooming and/or abuse through the sharing of sexual images or videos online under the age of 18; (b) were above the age of 16; and (c) lived in the UK.

### 2.2. Design

Qualitative semi structured interviewers were conducted with eight participants. A qualitative approach was important in exploring the novel research topic of long-term impact of OSA by providing the foundational understanding from individuals with lived experience. Thematic analysis was chosen as it allowed for the identification of patterns and themes within the data and provides a flexible interpretive approach. The research was underpinned by a critical realist epistemological position. This was considered appropriate given the importance of understanding the participants’ individual experiences but also acknowledging both participant and researcher subjectivity. This approach assumes that the data informs reality but does not mirror it and therefore requires interpretation (Willig, 2012). A critical realist position is also useful in allowing for discussion of the transferability of the study findings beyond the study sample, which is important in this emerging field (Fletcher, 2017). The consolidated criteria for reporting qualitative research (COREQ) checklist was consulted when reporting this research (Tong et al., 2007; Supplementary material).

Interviews were semi-structured and informed by a topic guide which asked about the nature of the participant’s unwanted online sexual experience, the impact of the experience on both their perceived self-esteem and mental health consequences, impact on relationships with family and friends, experience of disclosure and support received. Careful consideration was taken in developing the interview topic guide to ensure it was sufficiently flexible to allow for further exploration of the topics discussed by participants.

### 2.3. Procedures

Ethical approval was granted by the NHS Research Ethics Committee (19/NI/0167). Recruitment took place between January 2022 and February 2023, principally through three NHS CAMHS and SARC services. After establishing contact with the relevant service, the study was presented to clinical teams where staff identified potential participants. Similar procedures were followed when recruitment occurred through nation-wide charity organizations, Social media advertisements were also posted on Twitter, Instagram, Reddit and advertisements were posted on university bulletins. Social media advertisements contained the authors’ email addresses through which participants were able to express interest. The advertisements included the phrase ‘unwanted sexual experiences online’ to be mindful of the sensitive nature of the topic and the range of experiences that can be considered as OSA.

Participants who confirmed they would like to take part were given the option of completing the interview either in-person or remotely (online). A final sample of eight participants, all female aged 18 to 28 years consented to take part in the study. Following informed consent, interviews were conducted by author FS, audio-recorded and transcribed verbatim.

### 2.4. Data analysis

The transcribed interviews were uploaded to NVivo 12, which was used to support analysis. Thematic analysis was used to analyze the data (Braun and Clarke, 2013). Open-ended questions dictated the flow of the exchange and probing questions were used to aid further elaboration of responses. Following the familiarization of the data, the initial codes summarized the data content, and the codes were then grouped into broader central themes. An inductive reflexive approach was predominately used in the analysis. Semantic coding was followed for the development of the codes. The themes were discussed within the research team throughout the coding process until the final list of themes was reviewed, updated, and agreed. Interview transcripts were read multiple times by the author FS to allow familiarity with the data. Initial codes were generated from the transcripts and where then grouped into potential conceptual themes by FS and reviewed and refined by the broader research team. To ensure they reflected the source data and the aims of the research. Differences of opinion were resolved through discussion.

### 2.5. Reflexivity

A reflective log was kept documenting the overall experience of the interview, reflections on the topic guide, and emergent themes. All authors have research and clinical interest in developing interventions for individuals who have experienced OSA. FS also has expertise in developing policy interventions for OSA. We acknowledge that these experiences may affect the analysis and interpretation of the data. Additionally, we recognize that the similarities in age and gender of the participants and FS could have influenced the collection and interpretation of data. To minimize the impact of this, questions were presented in an open and neutral way, and participants were encouraged to explore both positive negative aspects of the topics discussed. The reflective log was also used to document how response influenced the researcher’s own views, which were held in mind during the analysis process.

## 3. Results

Interview length ranged from 35 to 60 min. Two interviews were conducted online and six in-person. All participants self-reported experiencing abuse through the taking or dissemination of sexual images or videos online. Five participants experienced online grooming, which for two participants involved coercion into viewing sexual content by an offender. Four of the online grooming experiences involved the non-consensual taking and the distribution of their sexual images. All participants who experienced online grooming met their offenders online, through either chat rooms, gaming platforms or social media. Two participants experienced non-consensual distribution of their sexual images. One participant had taken the images consensually; the images were then distributed non-consensually. One participant had experienced offline sexual abuse that was non-consensually recorded and distributed online. A further participant experienced being coerced into viewing sexual videos for the purpose of sexual gratification by their then partner. The experiences varied in length of time. For some, the abuse through sexual material was a one-time incident; for others, it lasted a couple of years. For those that experienced online grooming, the contact ranged from a couple of months to years. Participants were only asked if the experience occurred prior to the age of 18. Due to the retrospective nature of the interviews some participants did not recall their exact age at the time of the initial OSA exposure. However, participants did mention, the estimated years between the time from the occurrence of the initial OSA exposure and the interview taking place, which ranged from 1–20 years since either the abuse or the contact with the offender ended.

### 3.1. Key themes

**Figure 1:**
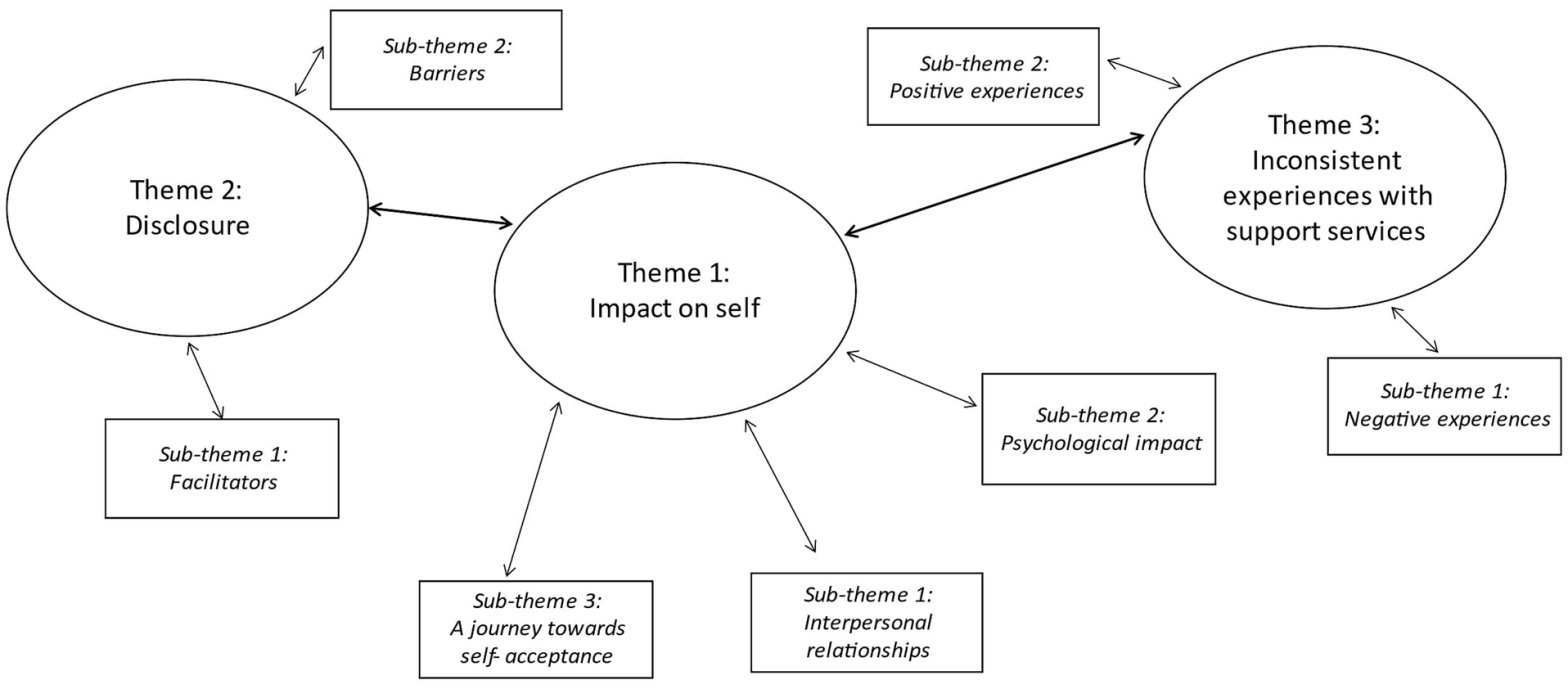
Thematic map.

Three interlinked themes were drawn from the findings: (1) the impact of OSA; (2) factors influencing disclosure; and (3) inconsistent experiences with support service. Themes and their sub-themes are displayed in a thematic map to show their relationships (Figure 1).

### 3.2. Theme 1: impact on self

Participants discussed difficulties within their interpersonal relationships, the psychological impact of their experience of OSA, their journey toward self-acceptance, and how these factors impacted their sense of self, both in terms of the immediate and longer-term impact.

#### 3.2.1. Sub-theme 1: difficulty in interpersonal relationships

##### 3.2.1.1. Immediate impact

Participants recalled isolating themselves from family and friends after the OSA experience. For some, this was because they were unsure to what extent their parents had known about the experience, or because they were uncomfortable discussing the experience with parents. Some participants isolated themselves for fear of either the offender finding them, being identified by others who have seen their sexual image, or because of feelings of self-hatred and shame. However, some participants expressed a desire for peer support shortly after the experience but had felt let down as their friends had not noticed that they were struggling and therefore did not offer support.

*“I was annoyed at everyone but especially one person because in my head I thought they should have done more to help even though I know they didn’t know everything I thought” (Female, 18).*

##### 3.2.1.2. Long-term impact

Participants struggled to find positive and supportive friendships after the OSA experience. For participants who had an online peer network prior to the OSA experience, they had become more cautious seeking out online friendships thereafter. For others, it was a question of trust within friendships. Participants described feeling worried about what friends might think, and judgments they might make about them if they disclosed the OSA experience:

*“I also think like ‘oh what do they actually think of me’ even though they have not made like an impression that they think any differently” (Female, 18).*

Seven participants discussed the impact on their romantic relationships. Three participants spoke about having continuous ‘on and off’ romantic relationships after the OSA experience. Participants recalled that after the contact had ended with the offender, they felt as if all their relationships would be abusive, and they believed that was how a normal, healthy relationship would be in the future. Participants discussed difficulties in setting boundaries, often because of the manipulation and coercion they had experienced, and how the experience increased their feelings of being sexualized in relationships. This included the expectation of sex and ‘giving in’ (sexually) to their partner’s wants. Additionally, participants discussed feelings of discomfort knowing that they will have to disclose the shared images to their future partners and being unsure how a future partner would respond:

*“If you don’t give them sex, they will go looking for sex elsewhere, and that would be your fault … so I let a number of my subsequent boyfriends push me sexually further than I might have gone otherwise” (Female, 28).*

*“kind of made me feel like, maybe like weaker in every relationship I have been in, except well, well every relationship I was in until the moment I was kind of like started rethinking everything” (Female, 24).*

#### 3.2.2. Sub-theme 2: psychological impact

The psychological impact that participants experienced was heavily intertwined with the previous sub-themes of interpersonal relationships. For example, feelings of anxiety were amplified and triggered in response to general questions they were asked by peers regarding where they live or what school they attend.

##### 3.2.2.1. Immediate impact

Participants described feelings of embarrassment and shame for sending the sexual images, and feelings of discomfort, confusion, and insecurity about the choices they had made. The shame and embarrassment participants felt at the time often contributed to feelings of self-blame:

*“It just, I wish I could change it, I know I can’t, I just, I just wish I could just speak to myself at 17 with the person I am now, and say you never ever needed to do, um, an it just-I just, I’m still insecure now probably because of that, because of the way that they saw me” (Female, 20).*

Participants also described feelings of embarrassment and shame for sending sexual images in the first place. Those who experienced online grooming described the difficulty coming to terms with the contact ending and felt as if they were processing the loss of a relationship. Participants described lacking motivation to do schoolwork or engage in hobbies in the weeks after the contact ended. Participants also described engaging in self-harm behavior following the OSA experience as a way of expressing the pain they were feeling once the contact had ended, or because of their struggle to feel understood either by friends or family or because they struggled with processing their own feelings about the experience:

*“I did start self-harming (…) but that was a way that I dealt with it because again I was made to feel like an outsider” (Female, 24).*

*“(the experience) can also like have reinforced something that was already there, I think I’ve always been a little bit like(…) maybe on the insecure side, and maybe it (the experience) kind of like reinforced that so I feel weaker in relationships and um, might like contribute to low mood and self-harm” (Female, 25).*

##### 3.2.2.2. Long-term impact

Participants described not recognizing the impact the experience had on their mental health until much later. For many, it was not until they had reflected on the experience months or years afterwards that they recognized the impact the OSA experience had on their mental health, including feelings of anxiety, discomfort, low mood, and low self-esteem. For participants who experienced online grooming, they only began to understand the abusive nature of the relationship years after the experience.

*“… definitely still struggle with the aftermath of all this and it comes in waves, kind of randomly sometimes, but I think I have a really strong understanding of a lot of the ways that I think and the way that I behave now” (Female, 25).*

*“I think it impacted me in other ways without me sitting there and ruminating about it so I can’t say that it helped me accept it that I hadn’t accepted it before, but I think if I’d never realised, I probably still would be in this cycle” (Female, 28).*

Most of the long-term issues discussed by participants related to ongoing feelings of self-blame and the struggle to accept they were not to blame. For participants who experienced online grooming, the betrayal and realization that the relationship was abusive was difficult and often required them to consciously overcome the feeling of self-blame. Participants who were asked for sexual images by the perpetrator knew the abuse was not their fault, but the words and tactics used by the perpetrator meant they felt like they had been an active agent in the abuse, which brought guilt around the choices they had made. Participants described feeling that it was ‘dumb’ to speak to strangers online and that as they actively initiated conversations, at some level they were at fault:

*“I want to say it was a sign of weakness-I didn’t want to show weakness in talking about it because it was my conscious decision to engage and have sort of conversation and relationships but as I say now looking back it wasn’t my fault” (Female, 19).*

Some discussed receiving mental health diagnoses such as anxiety, psychosis, obsessive-compulsive disorder, and post-traumatic stress symptoms. However, it was difficult for participants to delineate how specifically online abuse contributed to the specific mental health diagnosis they received. This was often because, even though participants brought up the OSA experience with their counselor, it was not a focal point in therapy:

*“I was having therapy on the hospital services for OCD and probably sort of mild depression but it was mainly sort of anxiety, (pauses) yea and they admitted that they weren’t really trained in that so we kind of talked about it … in different ways and I think I didn’t know what was helpful to me at that time, and he didn’t really know what to guess would be helpful so we kind of talked about but then we mainly just shifted towards OCD” (Female, 28).*

Some participants reflected on how the added element of online abuse significantly contributed to receiving a mental health diagnosis. For example, peers asked them general questions about where they lived or where they went to school, which contributed to them experiencing anxiety. Participants feared that once peers knew where they lived, they would become aware of the sexual images. Participants discussed how loss of control of the images, and image permanence, resulted on them feeling ‘exposed’ and ‘helpless’ with the knowledge that there was nothing they could do to remove the images:

*“it’s probably impacted me the most cause it still affected me now, cause I am also like paranoid about like who’s got pictures of me, like who’s looking at me, who’s giving me attention or whose not, and that is always replaying in my head” (Female, 20).*

#### 3.2.3. Sub-theme 3: a journey toward self-acceptance

When asked how the experience had impacted their sense of self, all participants described a long journey toward self-acceptance; sub-themes one and two were both woven into the discussion of self-acceptance. Participants described the social isolation from their interpersonal relationships (sub-theme one), struggles with their self-image after images had been distributed, and the how their overall perception of themselves impacted their mental health (sub-theme two).

##### 3.2.3.1. Immediate impact

Participants discussed their struggles with self-image after the OSA experience; they often described feeling ‘weak’ and ‘worthless’. Much of the discussion of self-image was intertwined with feeling sexualized during the experience and overlaps with the immediate impact participants described in sub-theme two. As an example, participants recalled that offenders repeatedly asking for sexual images often made them feel as if their self-worth was wrapped up in their body and appearance were only worth their body.

*“basically just became very closed off book almost, I was before that and I am now a very outgoing, I would describe myself as quite confident, um, a person but that just completely changed me as a person, I had no confidence, hated myself, doing anything that…where people were looking at me I would avoid” (Female, 24).*

*“I used to feel was like I can’t be sexualised like no one can see me as like pretty or like attractive, and now I am in a situation where people only want to use me for my body, so it’s like the total opposite … I was so like desensitised to showing my body, like you want a picture there you go, if that is all you want, that’s fine, that’s fine, take it” (Female, 20).*

The reactions of friends to discovering the participants sexual images also negatively impacted participant’s self-image. This included participants who recalled that peers had bullied and re-shared their sexual images. Participants would often believe the negative names that peers would call them, which contributed to their negative self-image and them ostracizing themselves from peers.

##### 3.2.3.2. Long-term impact

When reflecting on the experience years later, participants noted that the OSA experience was the ‘catalyst’ in how they viewed themselves and the image that they perceived others had of them. It was difficult for participants to build a positive self-image that was not tied to their appearance. The negative view of oneself contributed to difficulties in interpersonal relationships and personal struggles with mental health problems. Likewise, the subsequent negative self-image that developed over time resulted in participants avoiding posting images of themselves on social media or being in images in general:

*“… the catalyst of issues with self-image, understanding how I perceive myself, how others perceive myself, um, I think yea … that’s where it hit the most” (Female, 19).*

*“I wear baggy clothes a lot I am more comfortable in stuff that is not very revealing, I really struggle to be, I say complimented in inverted commas, but you know when you go out in the nightclubs and people pay attention, I don’t like that” (Female, 28).*

Participants described how the OSA experience made it difficult for them to identify with their culture or religion; it was difficult for some to accept that they had sent sexual images which did not align with cultural norms and expectations:

*“I was raised religious … the way I was raised, so that when anyone sort of speaks to you romantically their intention is to marry you, like they have pure intentions, you know they don’t mind if you don’t want to have sex, you don’t want to do things, so I was really naïve like I would speak to all these boys …it still haunts me now and stuff, um, of like me having this reputation especially in like the Asian community” (Female, 20).*

All participants described a journey of learning and growing in? confidence. Participants recognized confidence within themselves over time through feeling more protective over their body and establishing healthy boundaries around sex within relationships. Others expressed that connecting to religious groups was important in both fostering support and building a positive self-image. Participants described that, over time, feelings of self-blame around taking the images in the first place diminished.

### 3.3. Theme 2: factors influencing disclosure

Disclosure often involved a process of discovering that sexual images have been taken non-consensually and that their sexual images had been distributed online. For many, discovering that sexual images had been circulated online impacted their mental health, often bringing feelings of worry and anxiety, as reflected in theme1 one and sub-theme two. Some participants recalled feeling ashamed when their friends/family informed them that the images they had taken and shared with the offender appeared online, which subsequently negatively impacted their relationship with their family/friends. Participants also recalled how they had been aware that sexualized images of themselves had been taken, but completely unaware that these images had been uploaded and shared online, only discovering years later that this had taken place.

#### 3.3.1. Sub-theme 1: barriers in disclosure

Six out of the eight participants discussed that their own understanding of the experience influenced their ability to disclose. For these participants, it was not until they were much older and started talking about their experience that they understood the abusive nature of the OSA experience and its impact. For many, the process of realization involved developing an awareness of online grooming and images being disseminated online:

*“… it started hitting me when I started talking about it, I started fully processing it and realising what had actually happened, because then it was just, uh, saying, it without any emotional attachment to, just saying ‘oh this happened, and I don’t ‘know if it affected me ‘but after talking about it more and more I-that’s when I realised that it did” (Female, 19).*

*“I got a little bit older I did start to suspect that they could be on the internet … I started to really worry about what had happened to those pictures and videos and if anyone had seen them” (Female, 25).*

Participants described how the lack of physical contact/abuse led them to the perception that OSA was not a severe enough threshold to seek help. Participants said that they often did not mention their experience of OSA in therapy, or if they were talking about abuse, the focus was on contact abuse which felt like it had a more perceived tangible impact compared to online sexual experiences. As a result of not disclosing or talking about the OSA, many participants recalled not fully recognizing and understanding the severity of the experience until much later:

*“I watched a program where it was a documentary about a girl a similar thing had happened but she resulted in suicide but um, I was like’ oh this is familiar’, like yea, this happened and I didn’t even think about it in that way, um, so I can’t remember how old I was, about, maybe 19 or 20, probably before I actually re-evaluate” (Female, 28).*

Participants were hesitant to disclose to family and friends as they did not feel sharing their experience of OSA would be met with a non-judgmental and open conversation; avoiding disclosing to trusted others had a negative impact on these interpersonal relationships because participants felt they were keeping secrets or hiding a part of their life from family/friends. This created a distance in, and sense of isolation from, these relationships.

#### 3.3.2. Sub-theme 2: facilitators in disclosure

Two positive themes emerged from the data, one regarding facilitators to disclosure and one regarding the positive impact disclosing had on participants. For many, disclosing experiences of OSA to family/friends had been gradual. Many participants were more likely to be open with their romantic partners than other relationships they had.

*“I think I became gradually more open over time … I think at the beginning I didn’t particularly tell my family that I had friends online and stuff, and … then eventually I would tell them, oh yea I meet this person on the game … I have always been really open with my partners; I have been really open with everything that is going on” (Female, 24).*

The decision to disclose varied for participants; some participants felt comfortable and in a supportive and trusting environment to make the disclosure. Others were tired of keeping a secret or family members had noticed a change in behavior that then led them to disclose. Through discussing the experience, participants described feeling relieved and mentioning that the positive reactions after the disclosure helped them feel supported:

*“the people that do know, are people that I am really close to, and, who are, yea, it definitively brought us closer and have all been very compassionate and understanding” (Female, 25).*

Talking about the experience in an environment that felt non-judgmental, open, and trusting created more positive interpersonal relationships for participants and helped them to process and understand the impact the experience had on them. Some participants chose to first disclose the OSA experience to a friend that they had suspected experienced a similar incident. For others, participants broached the subject by asking friends whether the relationship they had been in was normal and friends indicating that it was not.

*“I trusted her really intrinsically; um I knew that she would believe me, and she also had no power” (Female, 25).*

### 3.4. Theme 3: inconsistent experiences with support services

Participants described two main services that were involved in providing support for OSA experiences: psychological therapy and crisis support lines. Seven out of the eight participants had received some form of therapy but not necessarily for the OSA experience itself; in some cases, participants received support for emotional or mental health difficulties without bringing up the OSA experience. Only two participants said they had received support for the OSA experience specifically.

#### 3.4.1. Sub-theme 1: negative experiences

Participants largely described negative experiences when seeking help for their OSA experience from support services, which, for most participants, impacted their overall processing of the experience. Participants described either not being able to access support, feeling misunderstood by a counselor, or being made to feel like the experience of OSA was their fault. These negative experiences of support in turn reinforced feelings of isolation and loss of control and reflected the experience of OSA itself:

*“It was but the way the counsellor spoke to me … I mean she obviously wanted to understand why I did it, which I explained to her, but then it was almost-she almost didn’t make me normal; she basically couldn’t understand why I did it, she made me feel like I did something really really wrong” (Female, 24).*

The negative experience of seeking support and the challenges that participants experienced in navigating the mental health system precluded further help-seeking:

*“I am not in therapy now, I feel like I should … just in general it’s really difficult with doctors here, it’s horrible. I have insurance and they still tell me that I need letter from a GP, and it takes a month to get that and then they say it is not their field of expertise, so they can’t give me a referral” (Female, 24).*

All participants described a gap in their understanding of the online element of abuse they experienced. For participants who did not seek therapy specifically for OSA, they struggled to talk about their online experiences because they felt that it was not understood by the person they were seeking support from. For those who did talk in therapy about the experience, the therapist acknowledged the experience but then did not explore this further or did not understand the nuance and subtlety of the impact:

*“The therapist was like ‘well I am sure that the police would just take them off the internet’ I was like ‘that is not how the internet works’ … that doesn’t make sense, and it did not make me very inclined to tell her any more about it, or to express more of my anxieties around the permanence of it to her” (Female, 25).*

*“I found them a bit kind of, um, they basically told me that they can’t really help me unless, I think they, they didn’t understand the idea of like because I interact with people a lot online, a lot of my friends are online friends” (Female, 24).*

#### 3.4.2. Sub-theme 2: positive experiences

Six participants described a positive experience of help-seeking or of receiving therapeutic support, though the support they received focused mainly on managing their mood and general mental health and well-being rather than exploring the OSA experience directly. For the two participants that received specific support for OSA, the relationship they formed with the service and the counselor were critical to feeling understood and heard. Through this process, participants recalled improvements in mood and feelings of panic they had experienced. Other participants described that feeling understood, and a non-judgmental environment, helped. One participant found it helpful when the counselor checked their own understanding of the OSA experience:

*“I just I think (the service) is really good and they are very – they are very good at you know, like talking to you and actually-actually getting what it is you are trying to say, um, they are—um they are not judgement or anything” (Female, 24).*

## 4. Discussion

This study sought to explore the short-and long-term impact of online grooming and abuse through the production or dissemination of sexual images by interviewing adult survivors of childhood OSA. To the authors’ knowledge, this is the first qualitative study asking adult survivors to reflect on the short-and long-term impact, including psychological impact, of a childhood OSA experience. This is also one of the few studies exploring the prolonged impact of OSA (Joleby et al., 2020b; Chiu and Quayle, 2022). Two main themes were developed from the data: (1) impact on self; and (2) factors influencing disclosure. We found that OSA experiences had a significant impact on participants’ sense of self and how they relate to friends, family, and romantic partners. While most participants described the impact of these experiences on their longer-term mental health, the support received for the OSA experience was inconsistent. Our findings corroborate those of other studies indicating that young people who have experienced OSA can struggle with long-term feelings of anxiety of images being shared online (Joleby et al., 2020a) in addition to more immediate impacts such as self-blame and low mood (Whittle et al., 2013a; Hanson, 2017; Joleby et al., 2020a).

A key contribution that this study makes to the existing literature is emphasizing the persistent impact of the taking and distribution of sexual images. Existing literature has extensively discussed the fear, worry and anxiety victim’s face when sexual images are distributed online (Quayle et al., 2012; Whittle et al., 2013b; Hamilton-Giachritsis et al., 2017) and the guilt that victims experience about their perceived ‘complicit’ participation in the abuse (Whittle et al., 2013a; Hamilton-Giachritsis et al., 2020a). Out findings show that these concerns and worries can be long-lasting and linger into adulthood, potentially influencing the social and psychological lives of OSA victims many years after the abuse. However, not all participants experienced negative long-term reactions to childhood experiences of OSA. For example, two participants described a neutral reaction to the non-consensual sharing of their images as they eventually came to view them as part of many sexual images that can be found online, a view that has been documented in other survivors in previous literature (Gewirtz-Meydan et al., 2018). These findings suggest that strategies that could promote a similar reframing around the person’s concerns and anxiety around permanence of their images in the digital space may help victims manage the distress OSA victims experience.

Our findings also revealed the prolonged impact on victims’ sense of self. Participants described struggling with feeling over-sexualized in subsequent social and romantic relationships. For some participants, this contributed to not wanting to take images with friends or wearing loose clothing to hide their bodies. Previous qualitative studies have also described victims’ struggles with self-image, body-image, self-esteem, and self-acceptance (Whittle et al., 2013a; Joleby et al., 2020b). These struggles often contributed to interpersonal difficulties, mainly with family/friends. The negative impact of OSA on interpersonal relationships described by our participants resonates with findings of previous qualitative studies (Leonard, 2010; Quayle et al., 2012; Hamilton-Giachritsis et al., 2017) and wider offline CSA literature (Davis and Petretic-Jackson, 2000; Fisher et al., 2017). However, this study has shown how the experience of OSA may also influence an individual’s vulnerability to enter in abusive and coercive relationships after the abuse. The manipulation, coercion and over-sexualization described by our participants all contributed to their struggle in understanding healthy boundaries long after the experience ended. This emphasizes the longer-term impact of the OSA experience on negative interpersonal relationships, especially in romantic relationships.

The negative mental health impact of OSA has previously been discussed within the OSA literature (Quayle et al., 2012; Whittle et al., 2013a; Hanson, 2017; Joleby et al., 2020b). This study contributes to the existing literature by highlighting the long-term struggles with mental health months and years after the experience. The manipulation and coercion that victims experience during online grooming can often result in victims feeling a sense of responsibility for their actions (Whittle et al., 2013a, 2015; Hamilton-Giachritsis et al., 2020a). The sense of responsibility for the abuse and feelings of self-blame are common themes discussed in both the OSA literature and the wider CSA literature base (Coffey et al., 1996; McElvaney et al., 2013; Kennedy and Prock, 2016). Further, existing literature on both OSA and contact CSA has highlighted that many children do not disclose the abuse because they were not aware of the abusive nature of the experience at the time (Whittle et al., 2013a; Hanson, 2017; Brennan and McElvaney, 2020; Joleby et al., 2020b). A distinct finding of this study is the length of time taken (sometimes years) for participants to understand the severity and impact of the experience. For many participants, due to the absence of ‘contact abuse’ in the experience they endured, it was difficult to understand the severity of the experience. Upon understanding the abusive nature of their relationships with perpetrators, participants noticed how the experience had negatively impacted their mental health in the long-term, including how it impacted their sense of self and interpersonal relationships.

Almost all participants received counselling or therapy either through private or public mental health services. However, when talking about their experiences with support services, most participants were not asked about their online world or relationships directly. Likewise, when speaking about their online lives, only a few participants had a positive experience in how their therapists responded to OSA disclosures. This reflects existing research that shows that assessment tools are limited in questions prompting about people’s online world, experiences, harms or relationships, let alone online abusive experiences (El-Asam et al., 2021). Further, qualitative research with practitioners has shown a lack of adequate training that provides confidence for practitioners when asking and responding about online harms (Hamilton-Giachritsis et al., 2020b; Dimitropoulos et al., 2022; Quayle et al., 2023). Our findings suggest that participants need a comfortable and safe therapeutic relationship when disclosing OSA experiences. Indeed, slowly easing into the conversation of online activities and prompting the conversation with more general topics is a key finding in literature offering guidance for practitioners in how to approach the topic of online harm (Biddle et al., 2022).

### 4.1. Strengths and limitations

The sample included a broad age range exploring the impact of OSA, allowing for a variety of views and experiences across the age range to be captured. A broad age range is also helpful in understanding the prolonged impact of OSA experiences and disclosing experiences. Additionally, the aim of the study was focused on understanding the impact of two types of OSA: online grooming and abuse through the production of sexual images or videos. Compared with the standard broader definition of OSA, this narrower definition allowed for the study findings to be directly applicable and not confounded with all types of OSA. Further, the qualitative design of the study allowed the nuances of the participants’ experiences and the variety of experiences to be highlighted. There are, however, some limitations that need to be considered. Our sample was all female, which is in line with existing qualitative studies on OSA experiences (Joleby et al., 2020b; Chiu and Quayle, 2022) and current OSA statistics that highlight the overrepresentation of females (Internet Watch Foundation, 2023). However, our findings are therefore only transferable to females who have experienced OSA and do not reflect OSA experiences in other gender categories. Additionally, the time between the interview and the end of each participants’ experiences varied considerably (1–20 years), which could have an effect on the findings relating to the long-term impact of the OSA experience. Further, although overall efforts were made to ensure methodological rigor of the study, member checking (Mays and Pope, 2000) could have improved the quality of the findings.

### 4.2. Clinical implications and future research

While most participants received some sort of counselling or mental health support in the years following the abuse, their online experiences were often unspoken, not acknowledged, or even dismissed. This finding underlines the importance of practitioners being trained in inquiring about online experience and harms and responding to disclosures of online sexual abuse (Martin, 2014; Lindenbach et al., 2021). Further, as the development of the different types of OSA is heavily interwoven with young people’s online experiences, it is recommended that support services are including a service-user engagement strategy with young people to continue to understand their interactions with the online world and update their assessment tools and interventions accordingly.

Future research should capture the impact of OSA experiences in individual’s who do not identify as female. Although current prevalence rates indicate that females are more at risk of OSA experiences, some reports show that those of ethnic minority and different sexual orientations may also have an increased vulnerability (Thorn, 2022). Future research should also develop and evaluate measures that specifically inquire about and assess for OSA during initial clinical assessments or in clinical history taking meetings. While most research on the impact of OSA is qualitatively based, which provides a valuable foundational understanding of the impact of OSA, future studies should adopt a quantitative methodological approach and measure the impact of OSA over time using a longitudinal study design following up a cohort of people who have had an OSA experience to measure their psychological symptoms over a period of time.

## Data availability statement

The datasets presented in this article are not readily available because to protect the anonymity of the participants. Requests to access the datasets should be directed to felipa.schmidt@postgrad.manchester.ac.uk.

## Ethics statement

The studies involving humans were approved by NHS Research Ethics Committee (19/NI/0167). The studies were conducted in accordance with the local legislation and institutional requirements. The participants provided their written informed consent to participate in this study.

## Author contributions

FS: Conceptualization, Methodology, Supervision, Writing – original draft, Data curation, Formal analysis, Project administration, Software, Validation, Visualization, Writing – review & editing. FV: Conceptualization, Methodology, Supervision, Writing – original draft. SB: Conceptualization, Methodology, Supervision, Writing – original draft, Formal analysis.
